# Supplementary material for: Empirical methods for controlling false positives and estimating confidence in ChIP-Seq peaks
Source: BMC Bioinformatics. 2008 Dec 5;9:523. doi: 10.1186/1471-2105-9-523 (PMC2628906; doi:10.1186/1471-2105-9-523)
Supplement: Additional File 1 — A variety of html documents from the USeq web site detailing the available applications, their best usage, output file type descriptions, command line menus, etc. [file 1471-2105-9-523-S1.zip › USeqUserGuides/JavaDoc.zip!/JavaDoc/util/bio/annotation/class-use/GenericFeature.html]

Uses of Class util.bio.annotation.GenericFeature


|  |  |  |
| --- | --- | --- |
| **Overview** \| **Package** \| **Class** \| **Use** \| **Tree** \| **Deprecated** \| **Index** \| **Help** | |  |
| PREV   NEXT | **FRAMES**    **NO FRAMES**     **All Classes** |  |


---


## **Uses of Class util.bio.annotation.GenericFeature**

| Packages that use GenericFeature | |
| --- | --- |
| **gata.geneGlyphs** | For representing annotation, scale bars, and generic tracks. |

Uses of GenericFeature in gata.geneGlyphs

| Methods in gata.geneGlyphs that return GenericFeature | |
| --- | --- |
| `GenericFeature` | `GenericGlyph.getGenericFeature()` |

Constructors in gata.geneGlyphs with parameters of type GenericFeature

`GenericGlyph(GenericFeature gf)`

---


|  |  |  |
| --- | --- | --- |
| **Overview** \| **Package** \| **Class** \| **Use** \| **Tree** \| **Deprecated** \| **Index** \| **Help** | |  |
| PREV   NEXT | **FRAMES**    **NO FRAMES**     **All Classes** |  |


---
